# Germline Variants Incidentally Detected via Tumor-Only Genomic Profiling of Patients With Mesothelioma

**DOI:** 10.1001/jamanetworkopen.2023.27351

**Published:** 2023-08-09

**Authors:** Owen D. Mitchell, Katie Gilliam, Daniela del Gaudio, Kelsey E. McNeely, Shaili Smith, Maria Acevedo, Meghana Gaduraju, Rachel Hodge, Aubrianna S. S. Ramsland, Jeremy Segal, Soma Das, Feighanne Hathaway, Darren S. Bryan, Sanjukta Tawde, Shelly Galasinski, Peng Wang, Melissa Y. Tjota, Aliya N. Husain, Samuel G. Armato, Jessica Donington, Mark K. Ferguson, Kiran Turaga, Jane E. Churpek, Hedy L. Kindler, Michael W. Drazer

**Affiliations:** 1 Section of Hematology/Oncology, Department of Medicine, The University of Chicago, Illinois; 2 Department of Human Genetics, The University of Chicago, Illinois; 3 Department of Pathology, The University of Chicago, Illinois; 4 Department of Radiology, The University of Chicago, Illinois; 5 Department of Surgery, The University of Chicago, Illinois; 6 Division of Hematology, Medical Oncology, and Palliative Care, Department of Medicine, University of Wisconsin, Madison

## Abstract

**Importance:**

Patients with mesothelioma often have next-generation sequencing (NGS) of their tumor performed; tumor-only NGS may incidentally identify germline pathogenic or likely pathogenic (P/LP) variants despite not being designed for this purpose. It is unknown how frequently patients with mesothelioma have germline P/LP variants incidentally detected via tumor-only NGS.

**Objective:**

To determine the prevalence of incidental germline P/LP variants detected via tumor-only NGS of mesothelioma.

**Design, Setting, and Participants:**

A series of 161 unrelated patients with mesothelioma from a high-volume mesothelioma program had tumor-only and germline NGS performed during April 2016 to October 2021. Follow-up ranged from 18 months to 7 years. Tumor and germline assays were compared to determine which P/LP variants identified via tumor-only NGS were of germline origin. Data were analyzed from January to March 2023.

**Main Outcomes and Measures:**

The proportion of patients with mesothelioma who had P/LP germline variants incidentally detected via tumor-only NGS.

**Results:**

Of 161 patients with mesothelioma, 105 were male (65%), the mean (SD) age was 64.7 (11.2) years, and 156 patients (97%) self-identified as non-Hispanic White. Most (126 patients [78%]) had at least 1 potentially incidental P/LP germline variant. The positive predictive value of a potentially incidental germline P/LP variant on tumor-only NGS was 20%. Overall, 26 patients (16%) carried a P/LP germline variant. Germline P/LP variants were identified in *ATM*, *ATR*, *BAP1*, *CHEK2, DDX41*, *FANCM*, *HAX1*, *MRE11A*, *MSH6*, *MUTYH*, *NF1*, *SAMD9L*, and *TMEM127*.

**Conclusions and Relevance:**

In this case series of 161 patients with mesothelioma, 16% had confirmed germline P/LP variants. Given the implications of a hereditary cancer syndrome diagnosis for preventive care and familial counseling, clinical approaches for addressing incidental P/LP germline variants in tumor-only NGS are needed. Tumor-only sequencing should not replace dedicated germline testing. Universal germline testing is likely needed for patients with mesothelioma.

## Key Points

**Question:**

What proportion of mesothelioma tumor profiling assays incidentally detect germline variants associated with hereditary cancer syndromes?

**Findings:**

In a case series of 161 unrelated patients with mesothelioma, 16% carried a pathogenic or likely pathogenic germline variant associated with hereditary cancer syndromes.

**Meaning:**

These findings suggest that mesothelioma patients may benefit from universal germline testing.

## Teaser

This case series study evaluates the prevalence of germline variants that were incidentally detected via tumor-only genomic profiling among patients with mesothelioma.

## Introduction

Mesothelioma is an aggressive cancer that principally affects the pleural and/or peritoneal cavities.^1^ The prognosis of mesothelioma is poor, with a median survival of only 18.4 months.^2^ Anatomic tumor location does influence prognosis, as individuals with peritoneal mesothelioma have longer survival compared with patients with pleural mesothelioma.^3^

Asbestos exposure is the major known risk factor for mesothelioma, but prior work has shown 12% of patients also carry germline pathogenic or likely pathogenic (P/LP) variants that further modify an individual’s lifetime risk of solid tumor development.^3,4,5,6,7^ Individuals with peritoneal mesothelioma are particularly likely to carry germline P/LP variants (25% of cases).^4^ Universal germline genetic testing, which is increasingly a standard of care in other cancers, is not yet routinely used in mesothelioma care.^8^ Clinical guidelines for mesothelioma recommend discussing the risks and benefits of germline genetic testing with patients who have personal or family histories that are suggestive of a hereditary cancer syndrome, particularly if these malignant neoplasms are associated with germline *BAP1* P/LP variants.^9^ Experiences with other tumor types have shown that universal genetic testing, as opposed to germline testing triggered by high-risk personal and/or family histories, increases the diagnosis of hereditary cancer syndromes.^10^

Germline *BAP1* P/LP variants are the most frequent and well-studied alterations in patients with mesothelioma who have increased cancer risk. Patients with mesothelioma and germline P/LP variants, especially in *BAP1*, have improved survival relative to patients without germline P/LP variants.^3,11^ Recognition of patients with germline *BAP1* P/LP variants may also help guide treatment decision-making, particularly in regard to the use of platinum-based therapies.^3,4,9^

Tumor-only next-generation sequencing (NGS) is increasingly being used in academic practices, where NGS can determine eligibility for clinical trials investigating targeted agents. The genes that are most frequently somatically altered in mesothelioma include *BAP1, CDKN2A, DDX3X*, *NF2,* and *TP53*.^12,13^ These same genes, when altered in the germline, are associated with hereditary cancer syndromes (*BAP1, CDKN2A, NF2,* and *TP53)* as well as developmental delays and/or disabilities (*DDX3X*).^9,14,15,16,17,18^ Recognizing the germline origin of these variants has important implications for the counseling and care of both the patient and their family members.^9^ Some P/LP variants detected via tumor-only NGS may be of germline origin. The association between tumor-only NGS and germline sequencing has not previously been performed for patients with mesothelioma.

Here, we analyzed a large series of patients with mesothelioma who underwent both tumor and germline sequencing. We determined the prevalence of incidental germline findings that were also detected via tumor-only sequencing.

## Methods

### Study Population

Patients with mesothelioma presenting to University of Chicago Medicine (UCM) were consented to an annually reviewed, institutional review board–approved protocol, from April 2016 to October 2021. The study followed the Strengthening the Reporting of Observational Studies in Epidemiology (STROBE) reporting guideline. After thorough discussion of the protocol, including risks and benefits, confidentiality, and voluntary nature of participation, and allowing the patient adequate time for consideration and asking questions, informed consent was obtained. This protocol allowed for biobanking (peripheral blood, saliva, and tumor) and germline sequencing. Trained interviewers used a standardized survey to collect a personal and family history of cancer from each patient. Primary (occupational or environmental) or secondary asbestos exposure (living with persons exposed to asbestos) was self-reported by patients. All exposures were classified as definite, probable, possible, or no known exposure. Demographic data, such as participant-reported race, were also collected, as patients with European ancestry have historically been overrepresented in most mesothelioma studies, increasing the importance of including data from non-European populations.

### Germline Variant Detection and Interpretation

DNA was extracted from peripheral blood mononuclear cells or saliva. DNA was sequenced with an 84 gene, research-based, NGS panel designed by the University of Chicago Genetic Services Laboratory to sequence the coding and flanking intronic regions of each gene (genes listed in eTable 1 in Supplement 1). All variants were analyzed by 2 independent reviewers (S.D. and D.G.) and interpreted according to the American College of Medical Genetics and Genomics and Association for Molecular Pathology consensus guidelines.^19^ P/LP variants, including nonsense, frameshift, splice site, missense variants, and large-scale genomic rearrangements with known moderate-to-high penetrance cancer susceptibility were reported. All germline P/LP variants were validated by Sanger sequencing, correlated with clinical and family history, and segregated in family members when possible. During the informed consent process, patients could opt in to receive disclosure of clinically relevant research results. Patients selecting yes were provided with clinical appointments with a physician and/or genetic counselor to discuss these results.

### Somatic Variant Detection and Interpretation

DNA was extracted from fresh frozen, paraffin embedded tumor tissue blocks. Somatic variants were identified using the UCM OncoPlus NGS panel, which sequences 1212 genes with median depths ranging from 360 × to 785 × coverage. eTable 1 in Supplement 1 contains 76 genes from the Oncoplus panel that are clinically reported.^20^

### Immunohistochemistry

BAP1 and PD-L1 staining was conducted in a CLIA-certified laboratory. The Santacruz, C4 monoclonal antibody was used for BAP1 staining and the Abcam 28.8 monoclonal antibody was used to evaluate PD-L1 staining. Percentage of cells positive for PD-L1 was calculated via the Tumor Percentage Score (TPS).

### Statistical Analysis

Fisher exact test and χ^2^ test were used to determine an association between patients with mesothelioma, the presence of germline variants, patient demographics, and tumor characteristics. A 2-sided *P *value of less than .05 was considered statistically significant. Data analysis was generated in Microsoft Excel, version 16, with the Real Statistics Resource Pack software, version 7.6, (Real Statistics) from August 2022 to March 2023.

## Results

### Study Population

**Figure 1.**
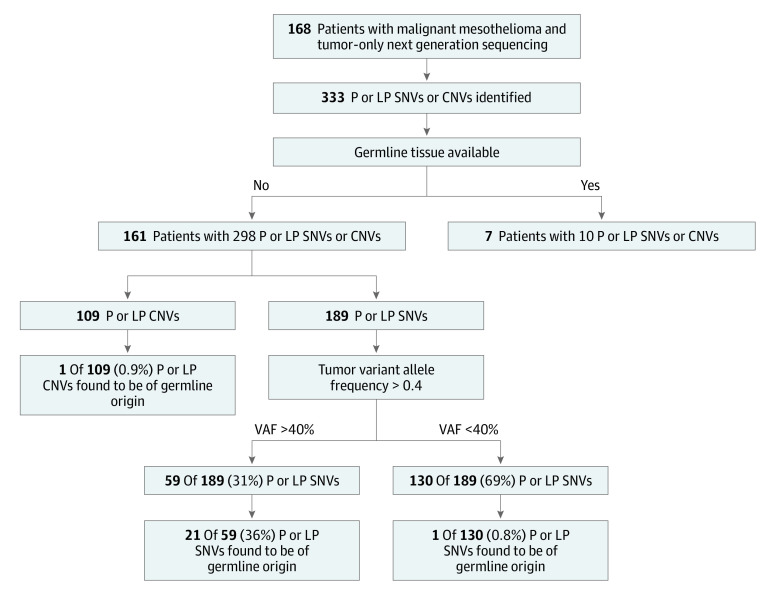
Flow Diagram of Patients in Mesothelioma Sequencing Cohort CNV indicates copy number variants; P, pathogenic; LP, likely pathogenic; SNV, single nucleotide variants.

Overall, 168 unrelated patients with mesothelioma who had tumor-only NGS were included. Of these patients, 161 (96%) had sufficient germline tissue available for sequencing (Figure 1). Of the 161 patients, 105 were male (65%) and the mean (SD) age was 64.7 (11.2) years. Most patients self-identified as non-Hispanic White (156 patients [97%]). Approximately 68% of all patients had pleural mesothelioma (109 patients), 28% had peritoneal mesothelioma (45 patients), and 4% had bicavitary disease or involvement of the tunica vaginalis (7 patients). Most patients had epithelioid mesothelioma (138 patients [86%]), 14 patients (9%) had biphasic, and 8 patients (5%) had sarcomatoid mesothelioma. Most patients did not have a personal history of second cancers (122 patients [76%]). Among the 39 patients (24%) with second malignant neoplasms, 12 had a history of skin cancer not otherwise specified secondary to a lack of family knowledge and/or medical records, 8 had prostate cancer, 7 had lymphoma, and 4 had thyroid cancer. A total of 114 patients (71%) had a first-degree family member with a malignant neoplasm (Table 1).

**Table 1.** Characteristics of Mesothelioma Patients and Tumors

Characteristic	Patients, No (%)		*P* value
	Patients with P or LP germline variants	Patients without P or LP germline variants	
Sex			
Female	9 (36)	47 (35)	>.99
Male	16 (64)	89 (65)	
Age at diagnosis, y			
<20	0	0	.92
20-29	0	2 (1)	
30-39	0	1 (1)	
40-49	1 (4)	7 (5)	
50-59	6 (24)	31 (22)	
60-69	9 (36)	51 (38)	
70-79	6 (24)	36 (26)	
>80	3 (12)	8 (6)	
Race and ethnicity			
Asian or South Asian	0	3 (2)	>.99
Black	0	1 (1)	
White, Hispanic	0	1 (1)	
White, non-Hispanic	25 (100)	131 (96)	
Asbestos exposure^a^			
Definite	10 (40)	52 (38)	.77
Probable	4 (16)	32 (24)	
Possible	8 (32)	41 (30)	
No known exposure	3 (12)	10 (7)	
No data	0	1 (1)	
Mesothelioma site			
Bicavitary	1 (4)	4 (3)	.61
Pericardium	0	0	
Peritoneum	9 (36)	36 (26)	
Pleura	15 (60)	94 (69)	
Tunica vaginalis	0	2 (1)	
Tumor histology			
Benign multicystic	0	1 (1)	.69
Biphasic	2 (8)	12 (9)	
Epithelial	23 (92)	115 (85)	
Sarcomatoid	0	8 (6)	
Personal history of other cancer			
Yes	12 (48)	27 (18)	.005
No	13 (52)	109 (81)	
Other cancers^b^			
Colon	0	2 (6)	NA
Lymphoma	3 (19)	4 (13)	
Prostate	2 (13)	6 (19)	
Kidney	2 (13)	0	
Skin, not otherwise specified	3 (19)	9 (29)	
Thyroid	1 (6)	3 (10)	
Other	5 (31)	7 (23)	
Cancer in first degree relative			
Yes	23 (92)	91 (68)	.01
No	2 (8)	45 (33)	

Abbreviations: LP, likely pathogenic; P, pathogenic.

^a^ Self-reported.

^b^ Some patients had multiple other cancers.

### Somatic and Germline P/LP Variants

Overall, 25 of 161 patients (16%) ultimately carried a P/LP germline variant (Figure 1 and eTable 5 in Supplement 1). Most patients (126 patients [78%]) with tumor-only NGS had a P/LP variant detected in a gene associated with a hereditary cancer syndrome. The positive predictive value (PPV) of a P/LP variant identified on tumor-NGS was 20% (25 of 126 patients). A total of 28 germline P/LP variants were identified. Three patients carried P/LP germline variants in 2 different genes. Most germline variants were in genes involved in DNA repair: *BAP1* (8 patients [29% of patients with a germline P/LP variant]), *CHEK2* (6 patients [21%]) and *ATM* (3 patients [11%]). Other genes with a germline P/LP variant identified in at least 1 patient included *ATR, DDX41, FANCM, HAX1, MRE11A, MSH6, MUTYH, NF1, SAMD9L*, and *TMEM127* (Table 2). Each patient with multiple P/LP germline variants carried a P/LP *BAP1* variant with germline P/LP variants in additional genes: *TMEM127* (patient 8), *HAX1* (patient 9), and *SAMD9L* (patient 10).

**Table 2.** Patients With a Germline Pathogenic/Likely Pathogenic Variant

Patient	Gene	Variant on germline panel	Variant on tumor panel	Tumor VAF, %	Interpretation^a^	Inheritance	IHC BAP1 status	History of other cancers	First-degree family history of cancer
1	*ATM*	p. ?	p.?	50	P	D	RE	Breast	Uterine
2	*ATM*	p.E1978*	p.E1978*	44	P	D	L	Thyroid	Prostate
3	*ATM*	p.E2052K	p.E2052K	48	LP	D	L	DLBCL	Colorectal
4	*ATR*	p.R443*	p.R443*	54	LP	D	L	No	Lymphoma NOS
5	*BAP1*	p.L573Wfs*3	p.L573Wfs*3	78	P	D	L	No	Mesothelioma
6	*BAP1*	p.L573Wfs*3	p.L573Wfs*3	43	P	D	L	Meningioma, BCC	BCC, breast, gilioblastoma, mesothelioma, testicular
7	*BAP1*	p.L573Wfs*3	p.L573Wfs*3	51	P	D	L	No	RCC, uterine
8	*BAP1*	p.Q260*	p.Q260*	52	P	D	L	Skin NOS	BCC, mesothelioma
9	*BAP1*	c.376_377del,p.?	c.376_377delp.?	NA	P	D	ND	Bladder, melanoma	Bladder, ovarian
10	*BAP1*	p.C91Wfs*35	p.C91Wfs*35	88	LP	D	L	No	Sarcoma
11	*BAP1*	Structural rerrangement^2^	Rearrangements	NA	P	D	L	No	Kidney
12	*BAP1*	p.R60*	p.R60*	46	P	D	L	RCC	Breast
13^b^	*CD36*	p.S113Ffs*20	NA	NA	P	R	R	No	Colorectal, prostate
14	*CHEK2*	p.I157T	p.I157T	70	LP	D	L	No	Breast, kidney NOS
15	*CHEK2*	p.W93Gfs*17	p.W93Gfs*17	49	P	D	L	Prostate	Colorectal
16	*CHEK2*	p.T476M	p.T476M	89	LP	D	L	No	None
17	*CHEK2*	p.T367Mfs*15	p.T367Mfs*15	42	P	D	L	Prostate, WM	Breast, prostate, thyroid
18	*CHEK2*	p.T367Mfs*15	p.T367Mfs*15	45	P	D	RE	Breast	Breast
19	*CHEK2*	p.I157T	p.I157T	49	LP	D	L	No	NHL, prostate
20	*DDX41*	p.R164W	p.R164W	46	LP	D	L	No	Lymphoma NOS
21^b^	*FANCM*	p.Q1701*	NA	NA	LP	R	RE	Paragan-glioma	Bladder
9^b^	*HAX1*	p.E31Kfs*54	NA	NA	P	R	ND	Bladder, melanoma	Bladder, ovarian
22	*MRE11A*	p.T408Nfs*49	p.T408Nfs*49	46	LP	R	ND	No	Colorectal
23	*MSH6*	p.F1088Lfs*5	p.F1088Lfs*5	16	P	R	R	No	Colorectal, skin NOS
24^b^	*MUTYH*	p.Y179C	NA	NA	P	R	L	CLL, BCC	None
25	*NF1*	p.P1650Lfs*48	p.P1650Lfs*48	47	P	D	R	RCC	None
10^b^	*SAMD9L*	p.Q1409Tfs*49	NA	NA	LP	R	L	No	Sarcoma
8^b^	*TMEM127*	p.L155*	N/A	NA	P	R	L	Skin NOS	BCC, mesothelioma

Abbreviations: BCC, basal cell carcinoma; CLL, chronic lymphocytic leukemia; D, dominant; DLBCL, diffuse large B-cell lymphoma; IHC, immunohistochemistry; L, lost; LP, likely pathogenic; ND, no data; NHL, non-Hodgkin’s lymphoma; NOS, not otherwise specified; P, pathogenic; R, recessive; RE, retained; VAF, variant allele frequency; WM, Waldenstrom macroglobulinmia.

^a^ Interpretation by The University of Chicago Genetic Services Lab.

^b^ Gene not sequenced on tumor NGS panel; all other genes names, variant detected in both germline and tumor tissue.

### Characteristics of Germline P/LP Variant Carriers

Most patients (15 patients [60%]) with P/LP germline variants had pleural mesothelioma, 36% (9 patients) had peritoneal mesothelioma, and 4% (1 patient) had bicavitary disease. Asbestos exposure was not significantly different between germline P/LP variant carriers and patients without germline variants (*P* = .77, Fisher exact test) (Table 1). Germline variant carriers were more likely to have a history of a second cancer (12 of 25 patients [48%]; *P* = .005; odds ratio [OR], 3.73; 95% CI, 3.19-4.24) as compared with patients without germline variants (27 of 136 patients [18%]). Most patients (11 of 12 patients [92%]) with P/LP germline variants and multiple cancers received a mesothelioma diagnosis concurrently or after the diagnosis of the other malignant neoplasm (eTable 2 in Supplement 1). The mean (SD) time between a first diagnosis of cancer and a mesothelioma diagnosis in patients with P/LP germline variants was 9.8 (14.5) years (eTable 2 in Supplement 1). Germline P/LP variant carriers were more likely to have at least 1 first-degree family member with a cancer diagnosis (23 patients [92%]; *P* = .01, Fisher exact test) than patients without a germline P/LP variant (91 patients [68%]) (Table 1).

### Pathologic Characteristics of Mesothelioma in Germline P/LP Variant Carriers

Overall, 23 of 25 germline P/LP variant carriers (92%) had epithelioid mesothelioma and 2 germline P/LP variant carriers had biphasic mesothelioma (8%). These proportions were similar to patients without germline variants (85% epithelioid and 9% biphasic or sarcomatoid; respectively, *P* = .69; OR, 0.94; 95% CI, 0.80-1.09) (Table 1). Most tumors in germline P/LP variant carriers (17 patients [68%]) lost BAP1 expression, which was similar to patients without germline P/LP variants (69 patients [51%]) (*P* = .28, Fisher exact test). Programmed death-ligand 1 (PD-L1) staining, which may be associated with immunotherapy response in mesothelioma, was similarly positive in patients with and without germline P/LP variants (56% and 45%, respectively; *P* = .38, Fisher exact test).^21,22^ Most patients with evaluable *CHEK2* germline P/LP variant carriers (5 of 6 patients [83%]) had tumors with BAP1 loss and positive PD-L1 staining (eTable 3 in Supplement 1).

### Plot of Somatic and Germline P/LP Variants

**Figure 2.**
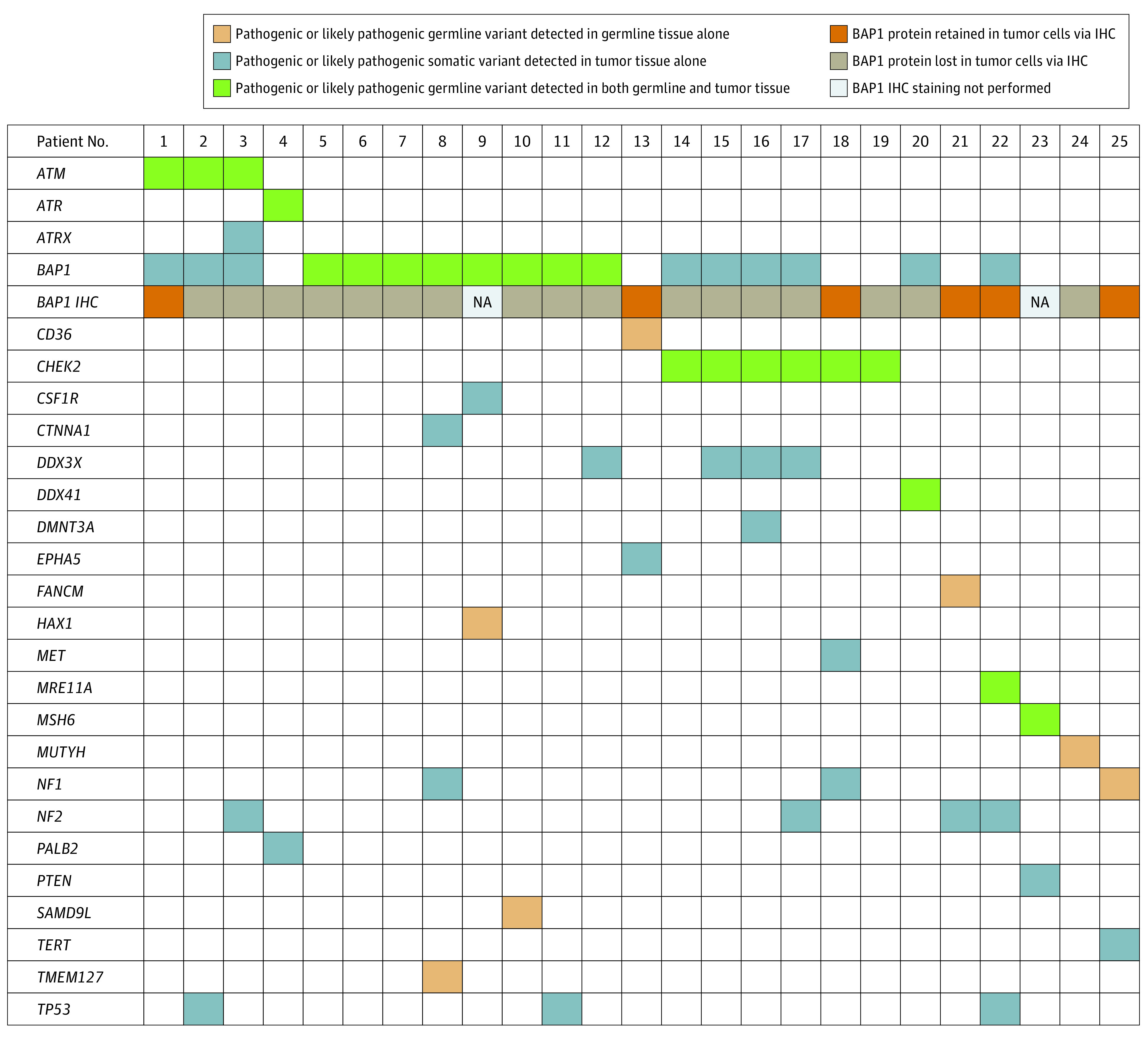
Pathogenic or Likely Pathogenic Variants in Patients With Germline Pathogenic/Likely Pathogenic Variants IHC indicates immunohistochemistry; NA, not applicable.

The tumor variant allele frequency (VAF) for incidental germline P/LP variants was greater than 40% in 19 of the 20 variants for which VAF was available (Table 2). Only 1 germline variant, a frameshift variant in *MSH6*, had a tumor VAF less than 40% (Table 2). The most frequent somatic variants in patients with germline P/LP variants were in *DDX3X* (4 patients), *NF2* (4 patients), and *TP53* (3 patients) (Figure 2). Three of 8 patients (38%) with germline P/LP variants in *BAP1* had second somatic hits in *BAP1*. The VAF of these second hits in *BAP1* ranged from 18% to 25%. All known germline P/LP variants in the patient cohort were incidentally detected via tumor-only NGS panels that sequenced the same genes (eTable 4 in Supplement 1). A small number of patients had P/LP germline variants detected via dedicated germline sequencing only, as the genes of interest (*CD36*, *FANCM*, *HAX1*, *MUTYH*, *SAMD9L*, *TMEM127*) were not sequenced by our institution’s tumor-only NGS panel.

## Discussion

Multiple groups have shown tumor-based NGS will incidentally detect germline P/LP variants in many patients with otherwise unremarkable personal and family histories.^23,24,25,26,27,28,29,30,31^ None of these studies have focused on patients with mesothelioma. Here, we demonstrate that 16% of unrelated patients (25 of 161) with mesothelioma carried P/LP germline variants associated with hereditary cancer syndromes. These variants were incidentally detected via tumor-only sequencing. Most patients (126 patients [78%]) in our cohort had variants detected via tumor-only NGS that warranted dedicated germline testing. The PPV of a potentially incidental germline P/LP variant on tumor-only testing was 20%. The high overall germline diagnostic yield in our cohort (25 patients [16%]) is similar to other tumor types in which universal germline genetic testing is now recommended, such as metastatic prostate cancer (12%-17%), epithelial ovarian cancer (18%-24%), and exocrine pancreatic cancer (15%).^32,33^ Universal genetic testing in all patients with cancer has been suggested as one approach to avoid missing any patients who harbor pathogenic germline variants. In 1 study^10^ of more than 2900 patients with cancer, 1 in 8 were found to be carrying a P/LP germline variant. Half of patients with P/LP germline variants did not meet guidelines for hereditary genetic testing. Given the high yield of patients with mesothelioma harboring a P/LP germline variant, which is similar to other cancer types where universal genetic testing is standard, it is reasonable to consider recommending universal germline screening in future mesothelioma treatment guidelines. Of note, only 1 patient in our cohort, a woman who had previously developed breast cancer in her 40s, would have met NCCN criteria for a hereditary cancer evaluation according to personal history.

We found patients with a family history of cancer had a higher likelihood of carrying a germline P/LP variant (92% vs 68%, respectively; *P* < .001) (Table 1). Despite this, we also identified germline variants in patients who did not have a family history of cancer. Given the importance of avoiding false negatives when deciding when to offer germline testing, family history alone is not a clinically acceptable surrogate for guiding germline testing decisions. We also found age at diagnosis, asbestos exposure, site of disease, and tumor histology were not associated with the presence or absence of a germline P/LP variant (Table 1). Tumor VAF has been proposed as a potential surrogate for germline variants.^34^ Once again, however, the importance of avoiding false-negative germline evaluations suggest that VAF alone should not be used as a surrogate for dedicated germline testing in patients with mesothelioma. Two patients in the germline P/LP variant cohort carried the *CHEK2* p.I157T, which has variable classifications of pathogenicity.^35,36^ According to the high prevalence of germline P/LP variants in patients with mesothelioma (16%), the high prevalence of variants on tumor-only NGS that warrant dedicated germline evaluation (78%), the inability to use tumor-based NGS as a germline assay, the clinical importance of identifying hereditary cancer syndromes, and the rapidly decreasing cost of NGS, we are likely approaching a point at which universal germline testing of patients with mesothelioma warrants consideration.

### Limitations

This study had limitations. One limitation is the 50 gene panel-based approach that we used for germline sequencing. This panel-based approach will have a lower diagnostic yield than more unbiased methods, such as whole exome or whole genome sequencing. However, panel-based sequencing is less expensive than more unbiased approaches, which improves the feasibility of implementing germline sequencing in a variety of clinical settings. Similar panel-based sequencing approaches have been used to establish the prevalence of germline variants in other tumor types.^33^ The high germline diagnostic yield (16%) in our cohort, despite our panel-based NGS approach and limited sample size, indicates that patients with mesothelioma represent a population that is at high risk for hereditary cancer syndromes regardless of sequencing approach and the size of panel used. We anticipate that the germline diagnostic yield in patients with mesothelioma will increase over time as more unbiased sequencing approaches are used. A second limitation is that our patient population was largely of European ancestry and was drawn from a single center, although the proportion of white patients in our retrospective study was similar to the proportion of patients with mesothelioma of European ancestry (93%) in larger data sets.^37^

## Conclusions

In this case series of 161 patients with mesothelioma, 16% had confirmed germline P/LP variants. Given the implications of a hereditary cancer syndrome diagnosis for preventive care and familial counseling, clinical approaches for addressing incidental P/LP germline variants in tumor-only NGS are needed. Universal germline testing is likely needed for patients with mesothelioma.

## Notes

Supplement 1. **eTable 1.** Genes Sequenced on the Germline and Somatic Panels **eTable 2.** Timeline of Multiple Cancer Diagnoses in Patients With Germline Variants **eTable 3.** Immunohistochemical Characteristics **eTable 4.** P/LP Somatic Variants Detected on Tumor NGS **eTable 5.** rsID and ExAC Frequency of Pathogenic or Likely Pathogenic Germline Variants

Supplement 2.
Data Sharing Statement

## References

[1] Cantini L, Hassan R, Sterman DH, Aerts JGJV. Emerging treatments for malignant pleural mesothelioma: where are we heading? Front Oncol. 2020;10:343. doi:10.3389/fonc.2020.00343 32226777 PMC7080957

[2] Faig J, Howard S, Levine EA, Casselman G, Hesdorffer M, Ohar JA. Changing pattern in malignant mesothelioma survival. Transl Oncol. 2015;8(1):35-39. doi:10.1016/j.tranon.2014.12.002 25749175 PMC4350634

[3] Hassan R, Morrow B, Thomas A, . Inherited predisposition to malignant mesothelioma and overall survival following platinum chemotherapy. Proc Natl Acad Sci U S A. 2019;116(18):9008-9013. doi:10.1073/pnas.1821510116 30975761 PMC6500142

[4] Panou V, Gadiraju M, Wolin A, . Frequency of germline mutations in cancer susceptibility genes in malignant mesothelioma. J Clin Oncol. 2018;36(28):2863-2871. doi:10.1200/JCO.2018.78.5204 30113886 PMC6804864

[5] Betti M, Casalone E, Ferrante D, . Germline mutations in DNA repair genes predispose asbestos-exposed patients to malignant pleural mesothelioma. Cancer Lett. 2017;405:38-45. doi:10.1016/j.canlet.2017.06.028 28687356

[6] Guo R, DuBoff M, Jayakumaran G, . Novel germline mutations in DNA damage repair in patients with malignant pleural mesotheliomas. J Thorac Oncol. 2020;15(4):655-660. doi:10.1016/j.jtho.2019.12.111 31887429 PMC7526793

[7] Pastorino S, Yoshikawa Y, Pass HI, . A subset of mesotheliomas with improved survival occurring in carriers of BAP1 and other germline mutations. J Clin Oncol. 2018;36(35):JCO2018790352. doi:10.1200/JCO.2018.79.0352 30376426 PMC7162737

[8] Daly MB, Pal T, Berry MP, ; CGC; CGC; LCGC; CGC; CGC. Genetic/familial high-risk assessment: breast, ovarian, and pancreatic, version 2.2021, NCCN clinical practice guidelines in oncology. J Natl Compr Canc Netw. 2021;19(1):77-102. doi:10.6004/jnccn.2021.0001 33406487

[9] Carbone M, Pass HI, Ak G, . Medical and surgical care of patients with mesothelioma and their relatives carrying germline BAP1 mutations. J Thorac Oncol. 2022;17(7):873-889. doi:10.1016/j.jtho.2022.03.014 35462085 PMC9233126

[10] Samadder NJ, Riegert-Johnson D, Boardman L, . Comparison of universal genetic testing vs guideline-directed targeted testing for patients with hereditary cancer syndrome. JAMA Oncol. 2021;7(2):230-237. doi:10.1001/jamaoncol.2020.6252 33126242 PMC7600058

[11] Baumann F, Flores E, Napolitano A, . Mesothelioma patients with germline BAP1 mutations have 7-fold improved long-term survival. Carcinogenesis. 2015;36(1):76-81. doi:10.1093/carcin/bgu227 25380601 PMC4291047

[12] Bueno R, Stawiski EW, Goldstein LD, . Comprehensive genomic analysis of malignant pleural mesothelioma identifies recurrent mutations, gene fusions and splicing alterations. Nat Genet. 2016;48(4):407-416. doi:10.1038/ng.3520 26928227

[13] Hung YP, Dong F, Torre M, Crum CP, Bueno R, Chirieac LR. Molecular characterization of diffuse malignant peritoneal mesothelioma. Mod Pathol. 2020;33(11):2269-2279. doi:10.1038/s41379-020-0588-y 32504035

[14] Goldstein AM. Familial melanoma, pancreatic cancer and germline CDKN2A mutations. Hum Mutat. 2004;23(6):630. doi:10.1002/humu.9247 15146471

[15] Hu C, Hart SN, Polley EC, . Association between inherited germline mutations in cancer predisposition genes and risk of pancreatic cancer. JAMA. 2018;319(23):2401-2409. doi:10.1001/jama.2018.6228 29922827 PMC6092184

[16] Asthagiri AR, Parry DM, Butman JA, . Neurofibromatosis type 2. Lancet. 2009;373(9679):1974-1986. doi:10.1016/S0140-6736(09)60259-2 19476995 PMC4748851

[17] Rocca V, Blandino G, D’Antona L, Iuliano R, Di Agostino S. Li-Fraumeni Syndrome: mutation of *TP53* is a biomarker of hereditary predisposition to tumor: new insights and advances in the treatment. Cancers (Basel). 2022;14(15):14. 35954327 10.3390/cancers14153664 PMC9367397

[18] Snijders Blok L, Madsen E, Juusola J, ; DDD Study. Mutations in DDX3X are a common cause of unexplained intellectual disability with gender-specific effects on Wnt signaling. Am J Hum Genet. 2015;97(2):343-352. doi:10.1016/j.ajhg.2015.07.004 26235985 PMC4573244

[19] Richards S, Aziz N, Bale S, ; ACMG Laboratory Quality Assurance Committee. Standards and guidelines for the interpretation of sequence variants: a joint consensus recommendation of the American College of Medical Genetics and Genomics and the Association for Molecular Pathology. Genet Med. 2015;17(5):405-424. doi:10.1038/gim.2015.30 25741868 PMC4544753

[20] Kadri S, Long BC, Mujacic I, . Clinical validation of a next-generation sequencing genomic oncology panel via cross-platform benchmarking against established amplicon sequencing assays. J Mol Diagn. 2017;19(1):43-56. doi:10.1016/j.jmoldx.2016.07.012 27836695

[21] Peters S, Scherpereel A, Cornelissen R, . First-line nivolumab plus ipilimumab versus chemotherapy in patients with unresectable malignant pleural mesothelioma: 3-year outcomes from CheckMate 743. Ann Oncol. 2022;33(5):488-499. doi:10.1016/j.annonc.2022.01.074 35124183

[22] Baas P, Scherpereel A, Nowak AK, . First-line nivolumab plus ipilimumab in unresectable malignant pleural mesothelioma (CheckMate 743): a multicentre, randomised, open-label, phase 3 trial. Lancet. 2021;397(10272):375-386. doi:10.1016/S0140-6736(20)32714-8 33485464

[23] Maani N, Panabaker K, McCuaig JM, . Incidental findings from cancer next generation sequencing panels. NPJ Genom Med. 2021;6(1):63. doi:10.1038/s41525-021-00224-6 34282142 PMC8289933

[24] Drazer MW, Kadri S, Sukhanova M, . Prognostic tumor sequencing panels frequently identify germ line variants associated with hereditary hematopoietic malignancies. Blood Adv. 2018;2(2):146-150. doi:10.1182/bloodadvances.2017013037 29365323 PMC5787862

[25] Catenacci DV, Amico AL, Nielsen SM, . Tumor genome analysis includes germline genome: are we ready for surprises? Int J Cancer. 2015;136(7):1559-1567. doi:10.1002/ijc.29128 25123297 PMC4303936

[26] Slavin TP, Banks KC, Chudova D, . Identification of incidental germline mutations in patients with advanced solid tumors who underwent cell-free circulating tumor DNA sequencing. J Clin Oncol. 2018;36(35):JCO1800328. doi:10.1200/JCO.18.00328 30339520 PMC6286162

[27] Scott AJ, Tokaz MC, Jacobs MF, Chinnaiyan AM, Phillips TJ, Wilcox RA. Germline variants discovered in lymphoma patients undergoing tumor profiling: a case series. Fam Cancer. 2021;20(1):61-65. doi:10.1007/s10689-020-00192-3 32504211 PMC7719097

[28] Meric-Bernstam F, Brusco L, Daniels M, . Incidental germline variants in 1000 advanced cancers on a prospective somatic genomic profiling protocol. Ann Oncol. 2016;27(5):795-800. doi:10.1093/annonc/mdw018 26787237 PMC4843184

[29] Green RC, Berg JS, Grody WW, ; American College of Medical Genetics and Genomics. ACMG recommendations for reporting of incidental findings in clinical exome and genome sequencing. Genet Med. 2013;15(7):565-574. doi:10.1038/gim.2013.73 23788249 PMC3727274

[30] Yushak ML, Han G, Bouberhan S, . Patient preferences regarding incidental genomic findings discovered during tumor profiling. Cancer. 2016;122(10):1588-1597. doi:10.1002/cncr.29951 26970385

[31] Gray SW, Hicks-Courant K, Cronin A, Rollins BJ, Weeks JC. Physicians’ attitudes about multiplex tumor genomic testing. J Clin Oncol. 2014;32(13):1317-1323. doi:10.1200/JCO.2013.52.4298 24663044 PMC3992721

[32] Hampel H, Yurgelun MB. Point/counterpoint: is it time for universal germline genetic testing for all GI cancers? J Clin Oncol. 2022;40(24):2681-2692. doi:10.1200/JCO.21.02764 35649230

[33] Uson PLS Jr, Samadder NJ, Riegert-Johnson D, . Clinical impact of pathogenic germline variants in pancreatic cancer: results from a multicenter, prospective, universal genetic testing study. Clin Transl Gastroenterol. 2021;12(10):e00414. doi:10.14309/ctg.0000000000000414 34620795 PMC8500569

[34] DeLeonardis K, Hogan L, Cannistra SA, Rangachari D, Tung N. When should tumor genomic profiling prompt consideration of germline testing? J Oncol Pract. 2019;15(9):465-473. doi:10.1200/JOP.19.00201 31509718

[35] Liu C, Wang QS, Wang YJ. The CHEK2 I157T variant and colorectal cancer susceptibility: a systematic review and meta-analysis. Asian Pac J Cancer Prev. 2012;13(5):2051-2055. doi:10.7314/APJCP.2012.13.5.2051 22901170

[36] Han FF, Guo CL, Liu LH. The effect of CHEK2 variant I157T on cancer susceptibility: evidence from a meta-analysis. DNA Cell Biol. 2013;32(6):329-335. doi:10.1089/dna.2013.1970 23713947

[37] Henley SJ, Larson TC, Wu M, . Mesothelioma incidence in 50 states and the District of Columbia, United States, 2003-2008. Int J Occup Environ Health. 2013;19(1):1-10. doi:10.1179/2049396712Y.0000000016 23582609 PMC4406225

