# Supplementary material for: Germline Variants Incidentally Detected via Tumor-Only Genomic Profiling of Patients With Mesothelioma
Source: JAMA Netw Open. 2023 Aug 9;6(8):e2327351. doi: 10.1001/jamanetworkopen.2023.27351 (PMC10413174; doi:10.1001/jamanetworkopen.2023.27351)
Supplement: Supplement 1. — eTable 1. Genes Sequenced on the Germline and Somatic Panels eTable 2. Timeline of Multiple Cancer Diagnoses in Patients With Germline Variants eTable 3. Immunohistochemical Characteristics eTable 4. P/LP Somatic Variants Detected on Tumor NGS eTable 5. rsID and ExAC Frequency of Pathogenic or Likely Pathogenic Germline Variants [file jamanetwopen-e2327351-s001.pdf]

Supplemental Online Content

Mitchell OD, Gilliam K, del Gaudio D, et al. Germline variants incidentally detected via tumor-only genomic profiling of patients with mesothelioma. *JAMA Netw Open*. 2023;6(8):e2327351. doi:10.1001/jamanetworkopen.2023.27351

eTable 1. Genes Sequenced on the Germline and Somatic Panels
eTable 2. Timeline of Multiple Cancer Diagnoses in Patients With Germline Variants
eTable 3. Immunohistochemical Characteristics
eTable 4. P/LP Somatic Variants Detected on Tumor NGS
eTable 5. rsID and ExAC Frequency of Pathogenic or Likely Pathogenic Germline Variants

This supplemental material has been provided by the authors to give readers additional information about their work.

© 2023 Mitchell OD et al. *JAMA Network Open.*

**eTable 1.** Genes Sequenced on the Germline and Somatic Panels									
GENES ON MESOTHELIOMA GERMLINE PANEL ONLY		GENES ON TUMOR PANEL ONLY				GENES ON BOTH PANELS			
ANKR26	NM_014915.2	*ABL1*	NM_005157.6	*HIST1H3C*	NM_003531.3	*APC*	NM_000038.5
BMPR1A	NM_004329.2	*AKT1*	NM_001382430.1	*HNF1A*	NM_000545.8	*ATM*	NM_000051.3
BRIP1	NM_032043.2	*ALK*	NM_004304.5	*HRAS*	NM_005343.4	*ATR*	NM_001184.4
CD36	NM_000072.3	*ARID1A*	NM_006015.6	*IDH1*	NM_005896.4	*BAP1*	NM_004656.3
EPCAM	NM_002354.2	*ARID2*	NM_152641.4	*IDH2*	NM_002168.4	*BARD1*	NM_000465.4
ERCC4	NM_005236.2	*ASXL1*	NM_015338.6	*ITPKB*	NM_002221.4	*BLM*	NM_000057.3
FANCB	NM_001018113.2	*AXL*	NM_021913.5	*JAK2*	NM_004972.4	*BRCA1*	NM_007294.4
FANCC	NM_000136.2	*B2M*	NM_004048.4	*KDR*	NM_002253.4	*BRCA2*	NM_000059.3
FANCD2	NM_033084.4	*BIRC3*	NM_001165.5	*KIT*	NM_000222.3	*CBL*	NM_005188.3
FANCE	NM_021922.2	*BRAF*	NM_001374258.1	*KMT2A*	NM_001197104.2	*CDH1*	NM_004360.4
FANCF	NM_022725.3	*CALR*	NM_004343.4	*KRAS*	NM_004985.5	*CDK4*	NM_000075.3
FANCG	NM_004629.1	*CBLB*	NM_170662.5	*MAP2K1*	NM_002755.4	*CKDN2A*	NM_000077.4
FANCI	NM_001113378.1	*CCND1*	NM_053056.3	*MAPK1*	NM_002745.5	*CEBPA*	NM_004364.4
FANCL	NM_018062.3	*CCND2*	NM_001759.4	*MDM2*	NM_002392.6	*CHEK1*	NM_001114121.2
FANCM	NM_020937.3	*CCND3*	NM_001760.5	*MET*	NM_000245.4	*CHEK2*	NM_007194.4
LIG4	NM_002312.3	*CDK6*	NM_001145306.2	*MLH3*	NM_001040108.2	*DDX41*	NM_016222.3
HAX1	NM_002382.4	*CSF1R*	NM_001288705.3	*MPL*	NM_005373.3	*ETV6*	NM_001987.5
MEN1	NM_130799.2	*CSF3R*	NM_000760.4	*MTOR*	NM_004958.4	*FANCA*	NM_000135.3
MUTYH	NM_001128425.1	*CTCF*	NM_006565.4	*MYC*	NM_002467.6	*FH*	NM_000143.3
NPAT	NM_002519.2	*CTNNA1*	NM_001903.5	*MYCN*	NM_005378.6	*GATA2*	NM_032638.4
PAX5	NM_016734.2	*CTNNB1*	NM_001904.4	*MYD88*	NM_002468.5	*IKZF1*	NM_006060.6
PMS1	NM_000534.4	*CUX1*	NM_181552.4	*NFE2L2*	NM_006164.5	*MLH1*	NM_000249.3
PMS2	NM_000535.6	*CXCR4*	NM_003467.3	*NOTCH1*	NM_017617.5	*MRE11A*	NM_005591.3
POLD1	NM_002691.3	*DAXX*	NM_001141969.2	*NOTCH2*	NM_024408.4	*MSH2*	NM_000251.3
RAD50	NM_005732.3	*DDR2*	NM_006182.4	*NPM1*	NM_002520.7	*MSH6*	NM_000179.3
RTEL1	NM_001283009.2	*DICER1*	NM_177438.3	*NRAS*	NM_002524.5	*NBN*	NM_002485.4
	NM_032957.5	*DNMT3A*	NM_022552.5	*PBRM1*	-	*NF1*	NM_000267.3
SAMD9L	NM_152703.4	*EGFR*	NM_005228.5	*PDGFRA*	NM_006206.6	*NF2*	NM_000268.3
SLX4	NM_032444.3	*EP300*	NM_001429.4	*PDGFRB*	NM_002609.4	*PALB2*	NM_024675.3
SRP72	NM_006947.3	*EPHA3*	NM_005233.6	*PIK3CA*	NM_006218.4	*POLE*	NM_006231.3
TERC	NR_001566.1	*EPHA5*	NM_001281766.3	*PIK3CB*	NM_006219.3	*POT1*	NM_015450.3
TMEM127	NM_017849.3	*ERBB2*	NM_004448.4	*PIK3R1*	NM_181523.3	*PTEN*	NM_000314.7
WRN	NM_000553.5	*ERBB3*	NM_001982.4	*PLCG2*	NM_002661.5	*PTPN11*	NM_002834.4
XRCC2	NM_005431.2	*ERBB4*	NM_005235.3	*PPP2R1A*	NM_014225.6	*RAD51*	NM_002875.4
XRCC3	NM_005432.4	*ERCC3*	NM_000122.2	*PTCH1*	NM_000264.5	*RAD51C*	NM_058216.3
		ESR1	NM_018010.4	*RAD21*	NM_006265.3	*RAD51D*	NM_002878.3
		EZH2	NM_004456.5	*RB1*	NM_000321.3	*RET*	NM_020975.5
		FAT3	NM_001367949.2	*SETBP1*	NM_015559.3	*RUNX1*	NM_001754.4
		FBXW7	NM_001349798.2	*SF3B1*	NM_006842.3	*SAMD9*	NM_017654.3
		FGFR1	NM_023110.3	*SMARCB1*	NM_003073.5	*SDHA*	NM_004168.4
		FGFR2	NM_000141.5	*SMC3*	NM_005445.4	*SDHAF2*	NM_017841.2
		FGFR3	NM_000142.5	*SMO*	NM_005631.5	*SDHB*	NM_003000.2
		FLT3	NM_004119.3	*SRSF2*	NM_001195427.2	*SDHC*	NM_003001.3
		FOXL2	NM_023067.4	*STAT3,*	NM_139276.3	*SDHD*	NM_003002.4
		GNA11	NM_002067.5	*STAT5B*	NM_012448.4	*SMAD4*	NM_005359.5
		GNAQ	NM_002072.5	*TET2*	NM_001146069.2	*STK11*	NM_000455.4
		GNAS	NM_000516.7	*TSC1*	NM_000368.5	*TERT*	NM_198253.2
		GRIN2A	NM_001134407.3	*TSC2*	NM_000548.5	*TP53*	NM_000546.6
		H3F3A	NM_002107.7			*VHL*	NM_000551.3

© 2023 Mitchell OD et al. *JAMA Network Open.*

eTable 2. Timeline of Multiple Cancer Diagnoses in Patients With Germline Variants

Pat #	First Cancer Dx	Age at Dx	Second Cancer Dx	Age at Dx	Third Cancer Dx	Age at Dx
1	**Peritoneal Mesothelioma**	57	Breast	58	NA	NA
2	Thyroid	30*	**Pleural Mesothelioma**	84	NA	NA
3	DLBCL	76	**Pleural Mesothelioma**	88	NA	NA
7	Melanoma	55	Bladder	59	**Peritoneal Mesothelioma**	65
10	Renal Cell Carcinoma	61	**Peritoneal Mesothelioma**	61	NA	NA
14	Prostate	60	**Pleural Mesothelioma**	60	NA	NA
16	Waldenstrom Macroglobulinemia	75	**Prostate**	76	**Pleural Mesothelioma**	77
17	Breast	46	**Pleural Mesothelioma**	74	NA	NA
20	Paraganglioma	75	**Pleural Mesothelioma**	75	NA	NA

*Approximation based on limited clinical information

© 2023 Mitchell OD et al. *JAMA Network Open.*

eTable 3. Immunohistochemical Characteristics

	Patients with Pathogenic or Likely Pathogenic Germline Genetic Variants		Patients without Pathogenic or Likely Pathogenic Germline Genetic Variants		p-Value
Immunohistochemistry Characteristics	*n*	[%]	*n*	[%]	
	25	16%	136	86%	
**Tumor BAP1 Status**					
Retained	6	24%	49	36%	0.295
Lost	17	68%	69	51%	0.277
No Data	2	8%	18	13%	
**Tumor PD-L1 Status**					
Positive	14	56%	61	45%	0.380
Negative	9	36%	49	36%	0.717
No Data	2	8%	26	19%	0.799
**% of PD-L1 Positive Tumor Cells**					
0-5%	7	50%	21	34%	0.450
6-20%	2	14%	9	15%	0.971
21-40%	2	14%	11	18%	0.891
41-60%	2	14%	16	26%	0.711
>61%	1	7%	4	7%	1.000
**PD-L1 Staining Intensity**					
Weak	5	36%	32	52%	0.506
Weak to Moderate	3	21%	10	16%	0.84
Moderate	3	21%	6	10%	0.65
Moderate to Strong	0	0%	6	10%	-
Strong	0	0%	2	3%	-
No Data	3	21%	5	8%	

	**Pathogenic or Likely Pathogenic Variants & Nonvariants in the Germline**							
	ATM		*BAP1*		*CHEK2*		OTHER	
Immunohistochemistry Characteristics	n	%	n	%	n	%	n	%
	3	17%	8	28%	6	33%	5	28%
**Tumor BAP1 Status**								
Retained	1	33%	0	0%	0	0%	2	40%
Lost	2	67%	7	88%	5	83%	2	40%
No Data	0	0%%	1	13%	1	17%	1	20%
**Tumor PD-L1 Status**								
Positive	2	67%	4	50%	6	100%	2	40%
Negative	1	33%	3	38%	0	0%	3	60%
No Data	0	0	1	13%	0	0%	0	0%

© 2023 Mitchell OD et al. *JAMA Network Open.*

**% of PD-L1 Positive Tumor Cells**										
		0-5%	0	0%	2	50%	3	50%	2	100%
		6-20%	0	0%	2	50%	0	0%	0	0%
		21-40%	1	50%	0	0%	1	17%	0	0%
		41-60%	1	50%	0	0%	1	17%	0	0%
		>61%	0	0%	0	0%	1	17%	0	0%
**PD-L1 Staining Intensity**										
Weak			0	0%	1	33%	2	40%	1	50%
Weak to Moderate			0	0%	0	0%	3	60%	0	0%
Moderate			2	0%	1	33%	0	0%	0	0%
Moderate to Strong			0	100%	0	0%	0	0%	0	0%
Strong			0	0%	0	0%	0	0%	0	0%
No Data			0	0%	1	33%	1	17%	1	50%

© 2023 Mitchell OD et al. *JAMA Network Open.*

eTable 4. P/LP Somatic Variants Detected on Tumor NGS

Patient	***Gene***	***Variant***	***Tumor VAF***	***Gender***	***Site of Disease***	***Histology***
1	*ATM*	c.4909_1G>T, p.?	50%	Female	Peritoneal	Epithelioid
	BAP1	c.740del, p.V247Gfs*2	31%			
2	*ATM*	c.5932G>T, p.E1978*	44%	Male	Pleural	Epithelioid
	BAP1	c.266del, p.N89Tfs*9	16%			
3	*ATM*	c.6154G>A, p.Q2052K	48%	Male	Pleural	Epithelioid
	ATRX	c.6201dup, p.P2068Tfs*5	32%			
	BAP1	c.659+1G>T, p.?	56%			
	NF2	c.448-2A>C, p.?	7%			
	TP53	c.532dup, p.H178Pfs*3	49%			
4	*ATR*	c.1327A>T, p.R443*	54%	Male	Pleural	Epithelioid
	PALB2	c.2587-1G>T, p.?	8%			
	TP53	Loss - Equivocal	-			
5	*BAP1*	c.1717del, p.L573Wfs*3	78%	Female	Peritoneal	Epithelioid
6	*BAP1*	c.1717del, p.L573Wfs*3	43%	Female	Pleural	Epithelioid
7	*BAP1*	c.1717del, p.L573Wfs*3	51%	Female	Peritoneal	Epithelioid
	BAP1	c.1949_1956delinsC, p.L650Pfs*3	18%			
8	*BAP1*	c.1330del ,p.T44Pfs*127	25%	Male	Bicavitary	Epithelioid
	BAP1	c.778C>T,e p.Q260*	52%			
	CTNNA1	c.468dup, p.V157Cfs*14	22%			
	NF1	c.1844del, p.K615Sfs*16	5%			
9	*BAP1*	c.376_377del, p.?	46%	Male	Peritoneal	Epithelioid
	BAP1	c.437+2A>T, p.?	23%			
	CSF1R	c.2276_2280del, p.L756Pfs*23	18%			
10	*BAP1*	c.272dup, p.C91Wfs*35	88%	Male	Peritoneal	Epithelioid
	BAP1	Loss	-			
11	*BAP1*	c.68-2A>G, p.?	24%	Female	Pleural	Epithelioid
	TP53	c.681dup, p.D228*	20%			
12	*BAP1*	c.178C>T, p.R60*	46%	Male	Peritoneal	Epithelioid
	DDX3X	c.968C>T, p.T323I	52%			
13	*EPHA5*	c.511dup, p.Y171Lfs*5	8%	Male	Peritoneal	Epithelioid
14	*BAP1*	c.37+9_122+60del, p.?	8%	Male	Pleural	Epithelioid
15	*BAP1*	c.354_358del, p.F118Lfs*6	21%	Male	Pleural	Biphasic
	CHEK2	c.277del, p.W93Gfs*17	49%			
	DDX3X	c.1056_1061del, p,M352_D354delins	34%			
16	*BAP1*	c.581-2del, p.?	76%	Male	Pleural	Epithelioid
	DDX3X	c.1438dup, p.R480Kfs*38	83%			
	NF2	c.114G>A, p.E38E	11%			

© 2023 Mitchell OD et al. *JAMA Network Open.*

17	*BAP1*	c.1321C>T, p.Q441*	9%	Male	Pleural	Epithelioid
	CHEK2	c.1100del, p.T367Mfs*15	42%			
	DDX3X	c.976C>T, p.R326C	20%			
	NF2	c.115G>A, p.E38E	11%			
18	*CHEK2*	c.1100del, p.T367Mfs*15	45%	Female	Pleural	Biphasic
	MET	c.3082+2T>C, p.?	24%			
	NF1	c.7188del, p.Y2398Tfs*20	9%			
19	-	None	-	Female	Peritoneal	Epithelioid
20	*BAP1*	c.256-1_259del, p.?	4%	Female	Pleural	Epithelioid
	DDX41	c.490C>T, p.R164W	46%			
21	*NF2*	c.1009C>T, p.Q337*	14%	Male	Pleural	Epithelioid
	TP53	Loss - Equivocal	-			
22	*BAP1*	c.2526-1G>A, p.?	55%	Female	Pleural	Epithelioid
	CDKN2A	Loss - Equivocal	-			
	MRE11A	c.1222dup, p.T408Nfs*49	46%			
	NF2	c.702_732del, p.G235Tfs*6	46%			
	TP53	c.528C>G, p.C176W	49%			
23	*MSH6*	c.3261dup, p.F1088Lfs*5	22%	Male	Pleural	Epithelioid
	PTEN	c.493G>C, p.G165R	49%			
24	*BAP1*	Rearrangement	-	Male	Pleural	Epithelioid
25	*CDKN2A*	Rearrangement	-	Male	Peritoneal	Epithelioid
	NF1	c.4947del, p.P1650Lfs*48	47%			
26	-	None	-	Female	Pleural	Epithelioid
27	*BAP1*	Loss	-	Male	Pleural	Epithelioid
28	*BAP1*	c.582del, p.W196Gfs*35	40%	Male	Pleural	Epithelioid
29	*MLH3*	c.3367C>T, p.Q1123*	49%	Male	Pleural	Epithelioid
	PTEN	c.121del, p.R41Dfs*13	26%			
	TP53	c.818G>T, p.R273L	43%			
30	-	None	-	Male	Peritoneal	Epithelioid
31	*BAP1*	Loss/Rearrangement	-	Male	Pleural	Epithelioid
	TP53	Loss	-			
32	*BAP1*	c.253C>A< p.Q85K	47%	Male	Pleural	Epithelioid
	DDX3X	c.546del, p.F182Lfs*39	54%			
33	*BAP1*	c.2012_2013insAA, p.Tyr671*	19%	Male	Pleural	Epithelioid
	NF2	c.616G>T, p.E206*	16%			
34	*CDKN2A*	Loss	-	Male	Pleural	Epithelioid
35	*BAP1*	c.376-25_376del, p.?	22%	Male	Peritoneal	Epithelioid
	BAP1	c.583_591del, p.P195_G197del	26%			
36	*BAP1*	c.1909_1910del, p.K637Vfs*5	21%	Male	Pleural	Epithelioid
37	*BRCA2*	c.8331+2T>C, p.?	22%	Female	Pleural	Biphasic
38	-	None	-	Female	Peritoneal	Epithelioid

© 2023 Mitchell OD et al. *JAMA Network Open.*

39	-	None	-	Male	Pleural	Epithelioid
40	*BAP1*	575del, p.D192Afs*39	14%	Male	Peritoneal	Epithelioid
41	*DDX3X*	c.1676T>A, p.L559H	32%	Male	Peritoneal	Epithelioid
42	*BAP1*	c.860C>G, p.S287*	16%	Male	Pleural	Epithelioid
43	*BAP1*	c.200A>G, p.D67G	17%	Male	Peritoneal	Epithelioid
	DDX3X	c.1423C>T, p.R475C	16%			
44	*TP53*	Rearrangement	-	Female	Pleural	Epithelioid
45	*BAP1*	Loss - Equivocal	-	Male	Pleural	Epithelioid
	BAP1	Rearrangement	-			
	CDKN2A	Loss	-			
	NF2	Loss	-			
46	*BAP1*	Rearrangement	-	Male	TVT	Epithelioid
47	*B2M*	c.246_247del, p.F82Lfs*7	36%	Male	Pleural	Epithelioid
	CDKN2A	c.341C>T, p.P114L	24%			
	NF2	c.1246C>T, p.R416*	34%			
	NRAS	c.182A>T, p.Q61L	29%			
	TERT	c.-124C>T, p.=	16%			
	TP53	c.396G>C, p.K132N	45%			
48	*NF2*	c.105_106insT, p.N36*	38%	Male	Peritoneal	Epithelioid
49	*ARID2*	c.4732C>T, p.Q1578*	43%	Male	Pleural	Epithelioid
	BAP1	Rearrangement	-			
	CDKN2A	Loss	-			
	CUX1	c.3327dup, p.D11110Rfs*65	9%			
50	*TP53*	c.524G>A, p.R175H	11%	Male	Pleural	Epithelioid
	TP53	c.818G>A, p.R273H	9%			
51	*FBXW7*	c.2065C>T, p.R689W	16%	Male	Pleural	Epithelioid
52	*BAP1*	c.1679dup, p.L561Pfs*6	66%	Female	Pleural	Biphasic
	CDKN2A	Loss	-			
53	*DDX3X*	c.1102_1106delinsa, p.D368Ifs*11	23%	Male	Peritoneal	Epithelioid
54	*NF2*	Loss - Equivocal	-	Male	TVT	Epithelioid
	STK11	Rearrangement	-			
55	*BAP1*	c.1360G>T, p.E454*	41%	Male	Pleural	Epithelioid
	NF2	c.276_299del, p.T93_F100del	44%			
56	*BAP1*	Loss	-	Male	Peritoneal	Epithelioid
	TP53	c.155_158dup, p.W53*	10%			
	TP53	Loss - Equivocal	-			
57	*CDKN2A*	c.332del, p.G111Afs*35	20%	Male	Pleural	Sarcomtoid
	TERT	c.-124C>T, p.=	22%			
	TP53	c.734G>C, p.G245V	26%			
58	*BAP1*	Loss - Equivocal	-	Female	BIcavitary	Epithelioid
	STK11	c.375-1C>T, p.?	28%			

© 2023 Mitchell OD et al. *JAMA Network Open.*

59	*BAP1*	Loss	-	Male	Pleural	Epithelioid
	CDKN2A	Loss - Equivocal	-			
	TP53	Loss - Equivocal	-			
60	*BAP1*	Rearrangement	-	Female	Peritoneal	Epithelioid
	WT1	c.1413_1432_55delinsT, p.?	29%			
61	*BAP1*	c.2057-2A>T, p.?	40%	Male	Peritoneal	Epithelioid
62	-	None	-	Male	Pleural	Epithelioid
63	*BAP1*	Loss	-	Female	Pleural	Epithelioid
64	*BAP1*	c.178C>T, p.R60*	21%	Male	Pleural	Epithelioid
	NF2	c.949G>T, p.E317*	22%			
	TP53	Loss - Equivocal	-			
65	*BAP1*	c.376-1G>A, p.?	22%	Female	Pleural	Epithelioid
	NF2	c.737del, p.P246Lfs*5	21%			
66	*BAP1*	Loss	-	Male	Peritoneal	Epithelioid
	PBRM1	c.899+2T>C, p.?	52%			
67	*BAP1*	c.2057073_2079del, p.?	18%	Male	Pleural	Epithelioid
	TP53	Loss - Equivocal	-			
68	*BAP1*	c.579_580dup, p.G194Vfs*38	28%	Male	Peritoneal	Epithelioid
69	*BAP1*	Loss - Equivocal	-	Female	Pleural	Epithelioid
	PBRM1	Rearrangement	-			
70	*BAP1*	c.1574_1599del, p.S525*	86%	Male	Pleural	Biphasic
	CDKN2A	Rearrangement/large-scale deletion	-			
	PTEN	c.388C>G, p.R130G	45%			
	PTEN	c.703dup, p.E235Gfs*8	9%			
71	*CDKN2A*	Loss - Equivocal	-	Female	Peritoneal	Epithelioid
	NF2	c.114+1G>A, p.?	11%			
72	*BAP1*	c.580+2T>C, p.?	20%	Female	Pleural	Sarcomtoid
	CDKN2A	Loss - Equivocal	-			
73	*ATM*	Rearrangement	-	Female	Peritoneal	Benign Multicystic
74	*TERT*	c.-124C>T, p.=	16%	Female	Pleural	Epithelioid
	TP53	Loss - Equivocal	-			
75	*CDKN2A*	c.173delinsCA, p.R58Pf*62	46%	Male	Pleural	Epithelioid
	KRAS	c.34G>T, p.G12C	55%			
	TP53	c.993+1G>A, p.?	45%			
76	*CHEK2*	c.920del, p.G307Efs*13	6%	Male	Pleural	Epithelioid
	NF2	Rearrangement/large-scale deletion	-			
77	*NF2*	c.51del, p.K17Nfs*8	22%	Male	Pleural	Biphasic
	TP53	c.403T>C, p.C135R	22%			
78	-	None	-	Female	Peritoneal	Epithelioid
79	*BAP1*	c.666del, p.Y223Tfs*8	22%	Male	Peritoneal	Epithelioid
	NF2	Rearrangement	-			

© 2023 Mitchell OD et al. *JAMA Network Open.*

80	*FAT3*	c.9193delinsTAT, p.R3065Yfs*15	6%	Male	Pleural	Epithelioid
	TP53	Loss - Equivocal	-			
81	*CDKN2A*	Loss - Equivocal	-	Male	Pleural	Epithelioid
	NF2	c.970C>T, p.Q324*	8%			
	TERT	c.-146C>T, p.=	16%			
82	*TERT*	c.-124C>T, p.=	6%	Female	Pleural	Sarcomtoid
83	*NF2*	c.855dup, p.N286*	40%	Male	Pleural	Epithelioid
84	*BAP1*	Loss	-	Male	Pleural	Epithelioid
	BAP1	Rearrangement	-			
	CDKN2A	Loss	-			
	FBXW7	c.1394G>A, p.R465H	25%			
85	*NF2*	c.1340+1G>T, p.?	17%	Female	Pleural	Epithelioid
	PBRM1	Rearrangement	-			
86	*ARID2*	c.3565C>T, p.Q1189*	15%	Male	Pleural	Sarcomtoid
	HNF1A	c.814C>T, p.R272C	15%			
	MSH6	c.3725G>A, p.R1242H	14%			
	NF2	c.193C>T, p.Q65*	18%			
87	-	None	-	Male	Peritoneal	Epithelioid
88	*BAP1*	c.438-1_452del, p?	6%	Male	Peritoneal	Epithelioid
	BAP1	c.86T>G, p.V29G	50%			
89	-	None	-	Female	Peritoneal	Epithelioid
90	*BAP1*	c.68-1G>C, p.?	22%	Male	Pleural	Epithelioid
	H3F3A	c.110A>G, p.K37R	18%			
91	*BAP1*	Loss	-	Male	Peritoneal	Epithelioid
	CDKN2A	Loss - Equivocal	-			
	CDKN2A	Rearrangement/large-scale deletion	-			
	STAG2	c.894-11_921del, p,?	37%			
	TP53	Loss - Equivocal	-			
92	*BAP1*	c.1828A>T, p.R610*	44%	Female	Pleural	Epithelioid
93	*BAP1*	Rearrangement	-	Female	Pleural	Epithelioid
	NF2	c.449_468del, p.T150Cfs*46	23%			
94	-	None	-	Male	Peritoneal	Epithelioid
95	-	None	-	Female	Pleural	Epithelioid
96	*BAP1*	c.255G>T, p.Q85H	18%	Female	Pleural	BIphasic
	TP53	c.375G>A, p.T125T	23%			
97	*BAP1*	c.1063C>T, p.Q355*	20%	Male	Pleural	Epithelioid
	CDKN2A	Rearrangement	-			
98	*BAP1*	Reaarrangement	-	Female	Pleural	Epithelioid
	NF2	c.1188del, p.K396fs*30	19%			
99	*NF2*	c.364-2A>C, p.?	24%	Male	Peritoneal	Epithelioid

© 2023 Mitchell OD et al. *JAMA Network Open.*

100	*BAP1*	c.1729+1G>A, p.?	60%	Female	Bicavitary	Epithelioid
101	*CDKN2A*	Loss	-	Male	Pleural	Epithelioid
	NF2	c.834_867del, p.K278Nfs*7	52%			
	NF2	Loss - Equivocal	-			
	TP53	Loss - Equivocal	-			
102	*BAP1*	c.71_87delinsACA, p.V24Dfs*40	21%	Male	Pleural	Epithelioid
103	*BAP1*	c.200A>G, p.D67G	21%	Male	Pleural	Epithelioid
104	*BAP1*	Loss	-	Female	Peritoneal	Epithelioid
	PBRM1	Loss	-			
105	*BAP1*	c.1941_1980delinsT, p.E647_F660delinsD	54%	Male	Pleural	Epithelioid
	NF2	c.655G>A, p. V219M	37%			
	TP53	c.154C>T, p.Q52*	37%			
106	*BAP1*	Loss	-	Female	Pleural	Biphasic
	BAP1	Rearrangement/large-scale deletion	-			
	CDKN2A	Loss	-			
	TP53	Loss - Equivocal	-			
107	*TP53*	c.578A>G, p.H193R	25%	Male	Pleural	Epithelioid
108	*TP53*	Loss - Equivocal	-	Male	Pleural	Epithelioid
109	*BAP1*	Loss	-	Female	Peritoneal	Epithelioid
	PBRM1	Loss	-			
110	*CDKN2A*	Loss	-	Male	Pleural	Epithelioid
	TP53	c.776A>T, p.D259V	56%			
111	*BAP1*	c.991_994del, .K331Pfs*3	38%	Male	Pleural	Epithelioid
	CDKN2A	Loss	-			
112	*BAP1*	c.1152_1198del, p.S384Rfs*3	10%	Male	Peritoneal	Epithelioid
113	*BAP1*	c.1638C>G, p.Y546*	8%	Male	Pleural	Epithelioid
	BAP1	c.203A>G, p.D68G	8%			
	CDKN2A	Rearrangement	-			
114	*TP53*	c.736A>G, p.M246V	22%	Male	Pleural	Epithelioid
115	*BAP1*	c.2186_2189delinsC, p.*729Qdelins*205	68%	Male	Pleural	Epithelioid
	CDKN2A	Loss	-			
	MDM2	Amplification	-			
116	-	None	-	Male	Pleural	Epithelioid
117	*TERT*	c.-124C>T, p.=	27%	Female	Pleural	Epithelioid
118	*CDKN2A*	Loss	-	Male	Pleural	Biphasic
119	-	None	-	Female	Pleural	Biphasic
120	*CDKN2A*	Rearrangement/Loss - Equivocal	-	Male	Pleural	Sarcomtoid
	NF2	c.970del, p.Q324Rfs*22	48%			
	TERT	c.-124C>T, p.=	55%			
121	*BAP1*	c.1911_1930delinsTTCCTGC, p.K637N*14	74%	Male	Peritoneal	Epithelioid
	TP53	Loss - Equivocal	-			
122	*PIK3CA*	c.1624G>A, p.E542K	26%	Female	Pleural	Epithelioid

© 2023 Mitchell OD et al. *JAMA Network Open.*

123	*TP53*	c.637C>T, p.R213*	37%	Male	Pleural	Epithelioid
124	*CDKN2A*	Loss - Equivocal	-	Male	Pleural	Sarcomtoid
	NF2	c.1396C>T, p.R466*	39%			
	TERT	c.-124C>T, p.=	24%			
	TP53	Loss	-			
125	-	None	-	Male	Peritoneal	Epithelioid
126	*BAP1*	c.687C>G, p.N229K	27%	Male	Pleural	Epithelioid
127	*CDKN2A*	Loss	-	Male	Pleural	Epithelioid
	TERT	Amplification - Equivocal	-			
128	*BAP1*	Loss	-	Female	Bicavitary	Epithelioid
	MLH3	c.3563C>G, p.S1188*	54%			
129	*CDKN2A*	Loss	-	Male	Pleural	BIphasic
130	*BAP1*	c.1177C>T, p.Q393*	33%	Male	Pleural	Epithelioid
	CDKN2A	Loss	-			
	NF2	Loss	-			
131	*ASXL1*	c.1772dup, p.T591*	6%	Male	Pleural	Epithelioid
	BAP1	Loss - Equivocal	-			
	CDKN2A	Loss	-			
	NF2	Rearrangement	-			
	NF2	Loss	-			
132	-	None	-	Male	Pleural	Biphasic
133	*BAP1*	c.1309_1311delinsT, p.L437Ffs*13	40%	Male	Pleural	Epithelioid
	CDKN2A	Loss	-			
	NF2	c.784C>T, p.R262*	66%			
134	*NF2*	c.1446+1G>A, p.?	24%	Female	Peritoneal	Epithelioid
135	*KDM6A*	Rearrangement	-	Female	Pleural	Epithelioid
	WT1	Rearrangement	-			
136	*BAP1*	Rearrangement	-	Female	Pleural	Biphasic
	MLH1	Rearrangement	-			
	TP53	c.380C>T, p.S127F	42%			
137	-	None	-	Female	Pleural	Epithelioid
138	*CDKN2A*	Loss	-	Female	Peritoneal	Epithelioid
	NF2	c.100dup, p.E34Gfs*15	29%			
139	-	None	-	Female	Peritoneal	Epithelioid
140	-	None	-	Male	Pleural	Epithelioid
141	-	None	-	Male	Pleural	Epithelioid
142	-	None	-	Male	Pleural	Epithelioid
143	*CDKN2A*	Loss	-	Male	Pleural	Epithelioid
	CS3FR	c.2259_2313del, p.R754Afs*8	21%			
	FBXW7	c.585-60_605del, p.?	17%			
	TP53	Loss - Equivocal	-			

© 2023 Mitchell OD et al. *JAMA Network Open.*

144	*CDKN2A*	Loss	-	Female	Pleural	Biphasic
	NF2	c.241del, p.V81Yfs*42	55%			
	TP53	c.666del, p.Y223Tfs*8	54%			
145	-	None	-	Female	Pleural	Epithelioid
146	*PBRM1*	Loss	-	Female	Peritoneal	Epithelioid
147	-	None	-	Male	Pleural	Sarcomtoid
148	*ARID2*	c.430C>T, p.Q144*	38%	Female	Pleural	Epithelioid
	TERT	c.-146C>T, p.=	24%			
	TP53	c.158G>A, p.W53*	14%			
	TP53	c.524G>A, p.R175H	6%			
	TP53	c.536A>G, p.H179R	12%			
149	*TP53*	c.1025G>C, p.R342P	8%	Male	Pleural	Epithelioid
150	*CDKN2A*	Rearrangement	-	Female	Pleural	Sarcomtoid
151	*ATM*	c.2239del, p.Q747Rfs*6	16%	Male	Peritoneal	Epithelioid
	BAP1	c.1730-1G>T, p.?	38%			
	NF2	c.389del, p.K130Rfs*44	33%			
152	*BAP1*	Loss	-	Female	Pleural	Epithelioid
	TP53	Loss	-			
153	*BAP1*	c.581-95_595del, p.?	25%	Male	Pleural	Epithelioid
154	*CDKN2A*	Loss	-	Female	Peritoneal	Epithelioid
	NF2	c.1126_1135del, p.S377Qfs*46	57%			
155	*CDKN2A*	Loss - Equivocal	-	Female	Pleural	Epithelioid
	NF2	Loss	-			
156	*MSH6*	c.3G>A, p.?	8%	Male	Peritoneal	Epithelioid
	TERT	c.-124C>T, p.=	5%			
157	*CDKN2A*	Loss	-	Female	Peritoneal	Epithelioid
	TP53	c.422G>A, p.C141Y	59%			
	TP53	Loss - Equivocal	-			
158	*BAP1*	Rearrangement	-	Male	Pleural	Epithelioid
159	*DDX3X*	c.1061A>T, p.D345V	29%	Male	Pleural	Epithelioid
160	-	None	-	Female	Bicavitary	Epithelioid
161	*BAP1*	c.553G>C, p.G185R	32%	Male	Pleural	Epithelioid
	CDKN2A	Loss - Equivocal	-			

© 2023 Mitchell OD et al. *JAMA Network Open.*

**eTable 5.** rsID and ExAC Frequency of Pathogenic or Likely Pathogenic Germline Variants			
Patient	Gene	rsID	ExAC frequency
1	*ATM*	rs756987454	-
2	*ATM*	rs587779852	7.00E-05
3	*ATM*	rs202206540	7.00E-05
4	*ATR*	-	-
5	*BAP1*	rs869025212	-
6	*BAP1`*	rs869025212	-
7	*BAP1*	rs869025212	-
8	*BAP1*	rs84622592	-
9	*BAP1*	-	-
10	*BAP1*	-	-
11	*BAP1*	-	-
12	*BAP1*	rs1253151209	-
13	*CD36*	-	-
14	*CHEK2*	rs17879961	1.00E-03
15	*CHEK2*	rs786203458	-
16	*CHEK2*	rs142763740	3.80E-04
17	*CHEK2*	rs555607708	1.82E-03
18	*CHEK2*	rs555607708	1.82E-03
19	*CHEK2*	rs17879961	4.10E-03
20	*DDX41*	rs142143752	1.40E-04
21	*FANCM*	rs147021911	1.30E-03
9	*HAX1*	rs764082747	1.20E-04
22	*MRE11A*	rs774440500	-
23	*MSH6*	rs267608708	-
24	*MUTYH*	rs34612342	1.62E-03
25	*NF1*	-	-
10	*SAMD9L*	-	-
8	*TMEM127*	rs886039439	-

© 2023 Mitchell OD et al. *JAMA Network Open.*
