# Supplementary material for: Germline Variants Incidentally Detected via Tumor-Only Genomic Profiling of Patients With Mesothelioma
Source: JAMA Netw Open. 2023 Aug 9;6(8):e2327351. doi: 10.1001/jamanetworkopen.2023.27351 (PMC10413174; doi:10.1001/jamanetworkopen.2023.27351)
Supplement: Supplement 2. — Data Sharing Statement [file jamanetwopen-e2327351-s002.pdf]

Data Sharing Statement

Mitchell. Germline Variants Incidentally Detected via Tumor-Only Genomic Profiling of Patients With Mesothelioma. *JAMA Netw Open*. Published August 09, 2023.
doi:10.1001/jamanetworkopen.2023.27351

Data
Data available: Yes
Data types: Other (please specify)
Additional Information: Other data will be made available
How to access data: Germline sequence variants - for upload to databases such as Clinvar as needed
When available: With publication

Supporting Documents
Document types: None

Additional Information
Who can access the data: Anyone requesting data from Clinvar team
Types of analyses: Public database - Clinvar
Mechanisms of data availability: With Investigator support
